# Clinical features of Sjögren’s syndrome patients with autoantibodies against interferons

**DOI:** 10.1186/s40169-018-0218-1

**Published:** 2019-01-03

**Authors:** Peter D. Burbelo, Sarah Browne, Steve M. Holland, Michael J. Iadarola, Ilias Alevizos

**Affiliations:** 1 Dental Clinical Research Core, NIDCR, NIH, Building 10, Room 5N106, 10 Center Drive, Bethesda, MD 20892-4410 USA; 2 Laboratory of Clinical Immunology and Microbiology, NIAID, NIH, Bethesda, USA; 3 Perioperative and Pain Section, Clinical Center, NIH, Bethesda, USA; 4 Sjögren’s Syndrome and Salivary Gland Dysfunction Unit, NIDCR, NIH, Bethesda, USA

**Keywords:** Anticytokine autoantibodies, Autoantibodies, Autoimmunization, Interferon-alpha autoantibodies, Sjögren’s syndrome

## Abstract

**Background:**

Sjögren’s syndrome (SS) is an autoimmune disease characterized by immune attack on the salivary and lacrimal glands. Given the known cytokine activation and type I interferon gene expression signature found in SS, we hypothesized that anticytokine autoantibodies might be detectable by Luciferase immunoprecipitation systems in some SS patients and correlate with clinical symptoms.

**Results:**

Luciferase immunoprecipitation systems was used to screen for serum anti-cytokine autoantibodies in 57 primary SS patients and 25 healthy volunteers. Autoantibodies were detected against GMCSF, interferon-γ, -α and, -ω in one, two, two and six patients with SS, respectively. None of the healthy volunteers showed anticytokine autoantibodies and none of the SS or control subjects showed autoantibodies against interferon-λ. One 51-year old female SS subject with the highest anti-interferon-α and -ω autoantibody levels had stable autoantibody levels over the course of a year. In vitro functional testing of serum autoantibodies from this subject demonstrated partially neutralizing activity for interferon-α signaling. Clinical information on this individual revealed a low focus score and high levels of unstimulated salivary flow, suggesting the possibility that interferon-α autoantibody neutralizing activity may have contributed to the milder sicca symptoms.

**Conclusion:**

Overall, these findings demonstrate that a subset of SS patients (16%) harbor autoantibodies against GMCSF, interferon-γ, interferon-ω, and interferon-α. These data support the observation that high levels of interferon-α autoantibodies may attenuate disease symptoms in SS.

## Background

Sjögren’s Syndrome (SS) is an autoimmune condition characterized by epithelial inflammation of the salivary and lacrimal glands, causing dysfunction of these exocrine glands [1]. The spectrum of symptoms seen in SS ranges from dry eyes and mouth to diverse, systemic extraglandular manifestations. Glandular inflammatory infiltrates, comprised of B-, T-, natural killer and other immune cells, along with cytokines, are key drivers of autoimmunity. Importantly, an activated type I interferon signature characterized by the up-regulation of many interferon inducible genes has been identified in SS patients through gene expression profiling of the salivary gland [2, 3], peripheral blood monocytes [4, 5], and plasmacytoid dendritic cells [5]. While low levels of interferon-α are found in the serum of SS patients, interferon-α-producing cells are enriched in the salivary gland, consistent with an increased local cytokine production [6]. In addition, other autoimmune conditions, including systemic lupus erythematosus (SLE), dermatomyositis, and psoriasis, also show an interferon signature [7–10]. In SLE patients, elevated interferon-α levels in blood correlate with disease flares [11–15].

Naturally occurring anticytokine autoantibodies are increasingly being linked to autoimmune-mediated immunodeficiency and other conditions [16–18]. In patients with pulmonary alveolar proteinosis (PAP), high levels of anti-GMCSF autoantibodies neutralize the activity of this cytokine and cause macrophage and neutrophil dysfunction leading to pulmonary pathology [19]. Similarly, patients with neutralizing anti-interferon-γ autoantibodies block signaling of this cytokine and are often associated with disseminated non-tuberculous mycobacterial (DNTM) infections [20–23]. Thymoma patients with elevated levels of autoantibodies against multiple cytokines, including interferon-α, anti-interferon-ω, and interleukin-12, have immune deficiency-like infections such as chronic mucocutaneous candidiasis, disseminated varicella zoster virus infection, and/or other opportunistic infections [24, 25]. Increasing evidence suggests that neutralizing anticytokine autoantibodies, in some cases, may also alter autoimmune disease activity. Notably, SLE patients harboring high levels of anti-interferon-α autoantibodies demonstrate less severe disease activity [26–29]. Furthermore, a subset of autoimmune polyendocrinopathy-candidiasis-ectodermal dystrophy patients exhibiting anticytokine autoantibodies did not develop type I diabetes [30]. Despite these findings, a mechanistic understanding of how anticytokine autoantibodies are generated in autoimmune diseases and their association with clinical symptoms remains poorly understood.

Luciferase immunoprecipitations systems (LIPS) utilizes light-emitting recombinant antigens to detect autoantibodies with high sensitivity and specificity in different autoimmune conditions [31] including SLE [28] and SS [32–36]. This is because LIPS is a fluid-phase immunoassay that presents autoantigens such as cytokines in solution, allowing them to adopt native structures. Numerous studies have shown LIPS to generate highly informative anticytokine autoantibody profiles in multiple human disorders [20, 25, 28, 30, 37–40]. In one study, LIPS was shown to strongly track the anticytokine autoantibodies observed by ELISA and protein array, but LIPS demonstrated autoantibody levels spanning a larger dynamic range of detection [30]. Based on the interferon-α gene expression signature found in SS, patients with this autoimmune condition were examined for anticytokine autoantibodies to determine if they correlated with clinical symptoms.

## Methods

### Healthy volunteer and SS subjects

All procedures performed in studies involving human participants were in accordance with the ethical standards of the institutional and/or national research committee and with the 1964 Helsinki declaration and its later amendments or comparable ethical standards. Sera from fifty-seven well-characterized patients diagnosed with primary SS and twenty-five healthy volunteers (HV) were evaluated as part of a natural history study under Institutional Review Board-approved protocol (IRB-D-0172) at the SS clinic of the National Institute of Dental and Craniofacial Research, National Institutes of Health, Bethesda, MD. The diagnosis of SS was established using the 2002 American-European consensus criteria [41]. These same serum samples were also used in a previous study for LIPS profiling of autoantibodies to Ro52, Ro60, and other autoantigens [32]. Most SS cases were from the time of diagnosis in the SS clinic.

In addition to the SS cases and healthy controls, selected positive control sera containing high levels of known anticytokine autoantibodies were collected under Institutional Review Board-approved protocols (NCT02190266) at the National Institute of Allergy and Infectious Diseases, National Institutes of Health, Bethesda, MD. These clinical samples included two PAP patients with high titer GM-CSF autoantibodies [42], two DNTM patients with high levels of interferon-γ autoantibodies [20], and two thymoma patients with levels of autoantibodies against interferon-α, interferon-λ, and interferon-ω [25]. Serum samples from these subjects were tested in parallel by LIPS as positive controls and in some cases for the study of serum anticytokine neutralizing activity.

Besides autoantibody data, other clinical information for the SS patients and volunteers included standardized tests for salivary flow rate, lacrimal gland function (Schirmer’s tests), and histopathological focus scores from minor salivary gland biopsy. The values for the focus score, a marker of clustered lymphocytic infiltrate in the salivary biopsy range from 0 (no infiltrate) to 12 (confluent). Rheumatoid factor (RF), extractable nuclear antigen (ENA), and antinuclear antibodies (ANA) were determined by ELISA in the Laboratory of Clinical Medicine, Clinical Center, NIH.

### Luciferase immunoprecipitations systems (LIPS) assays

Based on a previous study of anticytokine autoantibodies in SS [29], a select LIPS panel of five autoantigen targets was employed. The five cytokine targets included GM-CSF, IFN-γ, IFN- λ1, IFN-ω and IFN-α1 and have been previously described [20, 25, 28]. For LIPS autoantibody testing, serum samples were diluted 1:10 in assay buffer A (20 mM Tris, pH 7.5, 150 mM NaCl, 5 mM MgCl_2_, 1% Triton X-100), arrayed in 96 deep well microtiter plates, and tested as described [43]. Buffer blanks were used to monitor the performance and background binding activity of the LIPS assays. LUs were measured using a Berthold luminometer and all LU data were obtained from the average of at least two separate experiments.

### Analysis of anticytokine autoantibody neutralization activity

Using an established in vitro assay [20, 25, 29, 37, 44, 45], the neutralizing capacity of specific anti-interferon autoantibodies, were evaluated using control peripheral blood mononuclear cells (PBMC). In these experiments, PBMC were incubated in the presence of 10% healthy volunteer or patient sera and left unstimulated or stimulated with the cytokine recognized by the specific anticytokine autoantibody found in the serum samples. The PBMC were then fixed and permeabilized. To detect intracellular phosphorylation of the specific downstream Signal Transducer and Activator of Transcription-1 (STAT-1) the PBMC were immunostained using a monoclonal antibody to phospho-STAT-1 (BD Biosciences) and analyzed by flow cytometry. In the case of patients harboring interferon-α autoantibodies, cells were stimulated with interferon-α (1000 U/ml) and assessed for the corresponding interferon-α-induced pSTAT-1 in CD14+ monocytes. Data were collected using FACSCanto (BD Biosciences) and analyzed using FlowJo Version 9.1 software (TreeStar). Using this method, the amount of pSTAT production due to cytokine stimulation was extremely reproducible and sensitive to varying amounts of the cytokine added. Serum samples sera from the different subjects were then classified as non-neutralizing, partially neutralizing and neutralizing.

### Statistical and data analysis

GraphPad Prism software (San Diego, CA) was used for analysis of LIPS autoantibody data and for plotting values. For each test, LU were determined from the average of at least two separate measurements. Cut-off values were determined from the mean plus five standard deviations (SD) of the healthy volunteers and are indicated for each autoantibody test in the figures.

## Results

### Healthy volunteer and SS patient characteristics

A cohort of 25 healthy volunteers and 57 well-characterized patients with primary SS were tested for anticytokine autoantibodies. The age, gender, focus score, salivary flow, and autoantibody status are summarized in Table 1. The SS cases had an average age of 53 years (± 13) and 83% of them were women. The subjects with SS had a mean focus score of 4 (range 0 to 12) and showed an impaired mean unstimulated flow rates of 1.3 ml/15 min compared to 3.4 ml/15 min for the healthy volunteers. Approximately 65% of the SS subjects were seropositive for Ro52 and Ro60 autoantibodies.

**Table 1:** Characteristics of 57 patients with primary SS and 25 healthy volunteers (HV)

	SS (n = 57)	HV (n = 25)
Age, years (mean, ± SD)	53 (± 13)	48 (± 14)
Percent female	83%	60%
Unstimulated salivary flow, ml/15 min (mean, ± SD)	1.30 (± 2.30)	3.39 (± 3.39)
Focus score^a^ (mean, ± SD)	4 (± 4)	0 (± 1)
Schirmer’s test, mm/5 min (mean + SD)	5 (± 7)	14 (± 11)
Percent anti-Ro52 and anti-Ro60 seropositive	65%	0%

^a^Minor salivary gland biopsy and focus score data was available for all subjects. A focus score of 1 or greater is needed to show histopathological evidence of Sjögren’s Syndrome

### Detection of anticytokine seropositive SS patients

**Fig. 1:**
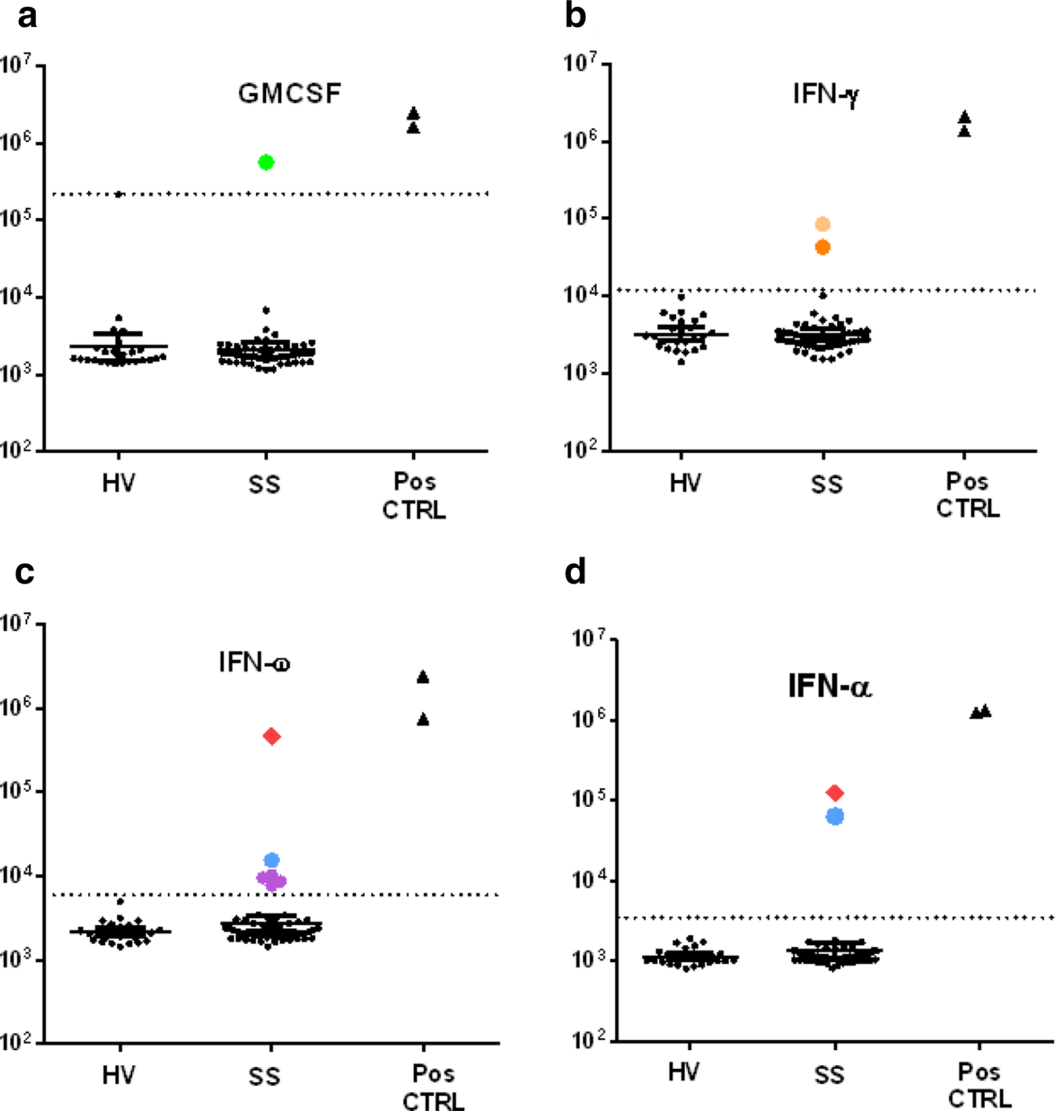
Detection of cytokine autoantibodies in SS. Using LIPS, autoantibodies against GMCSF (**a**), interferon-γ (**b**), -ω (**c**), and -α (**d**), were evaluated in 25 healthy volunteers (HV) and 57 Sjogren syndrome (SS) patients. Each circle on the graphs represents an individual subject. Autoantibody levels are expressed in LU. For each test, two appropriate positive serum from PAP, DNTB and thymoma patients were used as internal controls. The short solid lines for the HV and SS patients represent the geometric mean levels and 95% confidence interval for the autoantibody level in each group. The dashed lines represent the cut-off values defined as described in “Methods”. The SS patient (#56) with high levels of autoantibodies against interferon-α and interferon-ω is denoted by the red diamond

Based on previous studies in SLE and SS [28, 29], autoantibodies against a focused panel of five cytokine was examined by LIPS. Testing for autoantibodies against GM-CSF detected the two positive control PAP subjects with high autoantibody levels of 1,636,000 LU and 2,506,000 LU (Fig. 1a). In contrast, most of the healthy volunteers and SS cases had extremely low antibody levels with values less than 2000 LU (Fig. 1a). However, one healthy volunteer outlier and one SS patient had elevated GM-CSF autoantibody levels of 216,000 LU and 568,400 LU, respectively. A cut-off value derived from the mean plus five SD of the healthy volunteers revealed that only the one SS patient was seropositive for GM-CSF autoantibodies.

Autoantibodies against interferon-γ, -ω, -λ and -α were also evaluated. Testing of autoantibodies against interferon-γ revealed that the two positive, control DNTM cases had extremely high autoantibody levels of 2,078,000 LU and 1,380,000 LU (normal below 12,000 LU). Analysis of the cohort revealed that only two SS patients had statistically significant interferon-γ autoantibody levels (Fig. 1b). While markedly lower than the two DNTM positive controls, the two SS cases had autoantibody levels of 85,000 LU and 42,000 LU.

Autoantibodies against interferon-λ, interferon-ω and interferon-α were analyzed in the cohort along with two positive control thymoma patients with known high levels of autoantibodies against the three interferons. None of the healthy volunteers or SS subjects showed significant interferon-λ autoantibodies (data not shown). Six SS patients, but none of the controls, demonstrated statistically significant autoantibodies for interferon-ω (Fig. 1c). While five of the seropositives patients had interferon-ω autoantibody values that were quite low (just above the cutoff value), one SS subject had high levels of 465,800 LU. Analysis of interferon-α autoantibodies revealed that none of the 25 healthy volunteers had significant immunoreactivity above the cut-off value of 3500 LU. However, two of the 57 SS patients had anti-interferon–α autoantibodies with values of 124,800 LU and 64,070 LU, which were approximately tenfold lower than the thymoma positive controls (Fig. 1c). Notably, the two anti-interferon-α seropositive SS subjects were also seropositive for anti-interferon-ω autoantibodies. The subject with the highest anti-interferon-α levels, a 51-year old woman (#56), also had the highest anti-interferon-ω autoantibody levels (Fig. 1b, c). Further testing of an additional, available serial samples from this SS subject revealed that the anti-interferon–α autoantibody levels were roughly unchanged (i.e. 123,700 LU vs. 124,700 LU) during a one-year period.

### One SS patients contains partially neutralizing autoantibodies against interferon-α

**Fig. 2:**
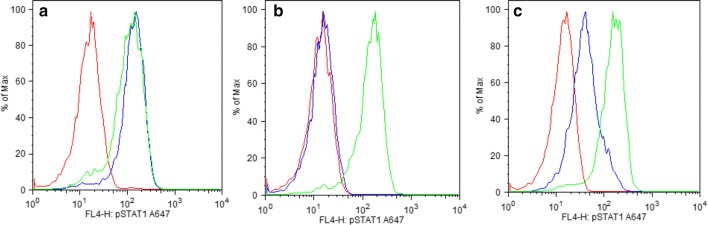
One SS patient contains partially neutralizing activity against interferon-α autoantibodies. PBMC were stimulated with interferon-α and interferon-γ in the absence and presence of serum. Phospho-STAT-1 monoclonal antibody followed by an anti-mouse-FITC secondary antibody was used for immunostaining. Each graph contains a red, blue, and green, line which represent cells unstimulated or stimulated by interferon-α, or interferon-γ, respectively. **a** HV control serum did not interfere with cell signaling by interferon-α and interferon-γ leading activation and phosphorylation of STAT-1. **b** In a thymoma patient serum, the presence of only anti-interferon-α autoantibodies block interferon-α binding therefore preventing phosphorylation. **c** SS patient #56, containing an intermediate level of serum anti-interferon-α autoantibodies can block some, but not all, interferon-α signaling activity leading to a partial block of phosphorylation

High levels of anticytokine autoantibodies may neutralize signaling as shown by in vitro assays [16]. Based on our previous LIPS studies showing that relatively high levels of anticytokine autoantibodies are required to observe blocking activity in vitro [20, 25, 37], only one subject, the 51-year old SS woman (#56) with relatively high levels of serum autoantibodies against interferon-α (and interferon-ω) was a candidate for functional testing of cytokine neutralizing activity. In these in vitro assays, exogenously added interferons activate cell signaling in PBMCs, resulting in phosphorylation of the STAT-1 transcription factor as assessed by flow cytometry assays. If neutralizing antibodies are present, phosphorylation of STAT-1 is blocked. As shown in Fig. 2a, in the presence of normal control human serum, the addition of interferon-α or interferon-γ readily led to phosphorylation of STAT-1. In contrast, serum from a thymoma patient with high anti-interferon-α autoantibodies completely blocked interferon-α-induced STAT-1 phosphorylation but had no effect on interferon-γ-induced STAT-1 phosphorylation (Fig. 2b). In the case of the serum from SS patient (#56), STAT-1 phosphorylation was partially inhibited in response to interferon-α stimulation, but this serum did not interfere with interferon-γ-induced STAT-1 phosphorylation (Fig. 2c). These results demonstrate that this SS patient (#56) contains serum autoantibodies that can partially neutralize interferon-α activity in vitro.

### Clinical correlates of anticytokine seropositive SS subjects

The nine SS subjects with anticytokine autoantibodies were also analyzed to determine whether these autoantibodies correlated with clinical symptoms, SSA (Ro52 and Ro60) and SSB (La) autoantibodies, focus score, unstimulated salivary flow, or other clinical data. The available clinical information from the nine anticytokine SS subjects revealed only a limited number of existing co-morbid conditions (Table 2). One of the SS cases with moderate levels of anti-interferon-α and anti-interferon-ω autoantibodies had pulmonary hypertension. Another subject with low-level anti-interferon-ω autoantibodies had anti-centromere autoantibodies and another had Hashimoto’s thyroiditis and lymphoma. Lastly, one of the two individuals with anti-interferon-γ autoantibodies had peripheral neuropathy (Table 2). The remaining four SS cases with cytokine autoantibodies, including patient #56, had only primary SS. Clinical laboratory results for the nine cytokine seropositive SS cases also demonstrated that all the subjects were seropositive for autoantibodies against ANA, ENA and RF (Table 2). Results the Schirmer test results, another diagnostic criterion for SS, revealed that seven of the negative subjects were positive for ocular inflammation (Table 2). Of note, two of the cytokine seropositive subjects, one harboring autoantibodies against GMCSF and patient #56 having high levels of autoantibodies against interferon-α were negative for ocular inflammation (Table 2).

**Table 2:** Clinical and laboratory features of anticytokine seropositive SS cases

Case	Cytokine autoantibodies	Clinical features	ANA	ENA	RF	Schirmer’s
13	Low IFN-ω		Pos	Pos	Pos	Pos
14	Low IFN-ω	Hashimoto’s thyroiditis, lymphoma	Pos	Pos	Pos	Pos
24	Mod IFN-γ		Pos	Pos	Pos	Pos
31	Low IFN-ω	Hypothyroidism, anti-ds DNA+, pANCA+	Pos	Pos	Pos	Pos
45	Mod IFN-α and -ω	Pulmonary hypertension	Pos	Pos	Pos	Pos
47	Low IFN-ω	Anti-centromere autoantibodies	Pos	Pos	Pos	Pos
56	High IFN-α and -ω		Pos	Pos	Pos	Neg
63	Mod IFN-γ	Peripheral neuropathy	Pos	Pos	Pos	Pos
69	High GMCSF		Pos	Pos	Pos	Neg

**Fig. 3:**
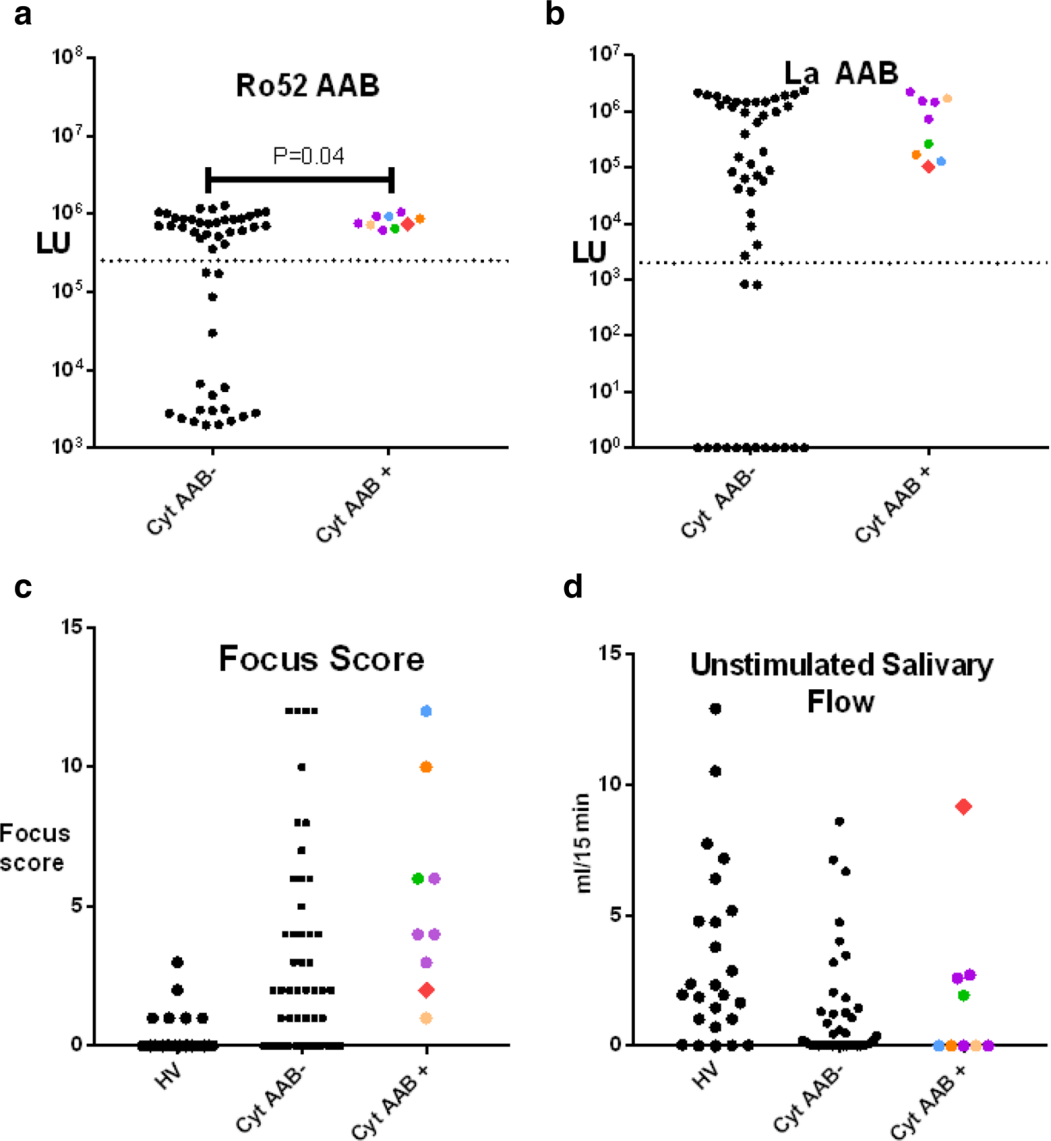
Clinical correlates of cytokine autoantibodies in SS. The SS cohort was stratified based on the 48 SS subjects with cytokine autoantibody seronegative status (Cyt AAB−), or 9 SS subjects with cytokine autoantibody seropositive status (Cyt AAB+). The color coding of the cytokine seropositive samples corresponds to their display in Fig. 1. The SS subject (#56) with partially neutralizing autoantibodies to interferon-α is shown by the red diamond. **a** Ro52 and **b** La autoantibodies were measured by LIPS and analyzed. The dotted line is based on cut-off value from HV. Mann–Whitney U test was used for statistical analysis. **c** The focus score, a histological marker assessment of the amounts of inflammatory cells in the salivary gland biopsy and **d** unstimulated salivary flow for the subjects is shown for the HV, Cyt AAB− and Cyt AAB+

Examination of the autoantibody status against SS-specific autoantigens, Ro52, Ro60 and La, showed that all nine anticytokine cases were seropositive for these three autoantigens (Fig. 3a, b and data not shown). Only the Ro52 autoantibody levels were higher (*p *= 0.03) in the anticytokine seropositive SS subjects as compared to the SS cases seronegative for anticytokine autoantibodies (Fig. 3a). Analysis of focus score, another key element of the diagnostic criterion for SS measuring inflammation in the salivary gland biopsy, revealed that the anticytokine seropositive SS subjects had focus score values ranging from 1 to 12 (Fig. 3c). While many of the anticytokine seropositive SS cases showed very high focus scores, no statistical difference (*p *= 0.059) was found between the SS subjects with and without cytokine autoantibodies. Interestingly, two subjects, the SS patient with partially interferon-α (#56) and another SS subject with moderate interferon-γ autoantibodies, showed a low focus score of 2 and 1, respectively (Fig. 3c). Lastly, inspection of the unstimulated salivary flow in the cohort revealed that most of the SS cases had impaired saliva production with flow values less than 2.6 ml/15 min. No statistical difference in flow rate was found between the SS cases with and without cytokine autoantibodies (Fig. 3d). However, patient #56 was the remarkable outlier exhibiting a very high unstimulated saliva flow of 9.2 ml/15 min, a value resembling that of the healthy controls.

## Discussion

While high levels of anticytokine autoantibodies are often identified in the setting of opportunistic infection [16, 17], here we report that 16% (9/57) of our SS cohort demonstrated statistically significant levels of autoantibodies against one or more cytokines, including GM-CSF and interferon-α, -γ, and -ω. Despite employing a different immunoassay for cytokine autoantibody detection, the prevalence of anticytokine autoantibodies is in general agreement with a recent larger study examining several different autoimmune diseases, including SS [29]. The finding that one SS patient showed autoantibodies against GM-CSF and two others harbored autoantibodies against interferon-γ are consistent with the activation of these pathways in SS [4]. Particularly intriguing was the high prevalence of autoantibodies to type I interferons. Similar to our previous findings in SLE [28], interferon-ω autoantibody seropositivity in the SS cohort showed the highest prevalence (11%; 6/57), suggesting this cytokine may play an important role in driving immune dysfunction in both autoimmune diseases. It is important to point out that interferon-ω is historically listed as separate interferon subtype, but structurally belongs it to subfamily of interferon-α [46]. Consistent with this observation, two of the interferon-ω subjects were seropositive for interferon-α autoantibodies. While interferon-α1 is known to be overexpressed within the salivary gland of SS patients [6], the exact tissue source of the multiple different cytokines, including interferon-ω, and interferon-α, driving autoantibody production in SS is not known. The finding that all nine SS patients with seropositive anti-cytokine autoantibodies also harbored additional autoantibodies against multiple other autoantigens including ANA, RF, Ro52, Ro60, and La, highlights the heightened B cell activation seen in the individuals. Since not all SS patients seropositive for these SS-related autoantibodies were seropositive for anticytokine autoantibodies, additional factors such as the timing/stage of SS, HLA differences, or concurrent infection by certain pathogens, are likely to play a role.

A study of rheumatoid arthritis, SS, and SLE patients found that SLE patients had the broadest and highest range of serum anticytokine autoantibodies [29]. In SLE, interferon-γ autoantibodies were found to correlate with the most severe disease activity. In our SS cohort, one of two anti-interferon-γ autoantibody seropositive cases had peripheral neuropathy. In addition, several of the anti-interferon-ω seropositive cases had Hashimoto’s thyroiditis and lymphoma, pulmonary hypertension, and anti-centromere autoantibodies. Due to the anticytokine autoantibody heterogeneity and the relatively small size of our cohort, a larger cohort of samples is needed to determine whether these anticytokine autoantibodies are associated with any specific sets of clinical findings.

SLE is another autoimmune disease characterized by activation of the type I interferon system [47]. Multiple studies have demonstrated that SLE subjects harboring high levels of anti-interferon-α autoantibodies, but not anti-interferon-ω autoantibodies, were associated with more clinically quiescent disease activity [26–29]. In our cohort, two SS cases exhibited autoantibodies against interferon-α, yet only one patient had relatively high levels of anti-interferon-α autoantibodies. Consistent with this observation, in vitro testing revealed that this SS subject (#56) had partially neutralizing serum autoantibodies to interferon-α. Although only serum was tested for anticytokine neutralizing activity, it is possible that high levels of interferon-α autoantibodies are present in the salivary gland. This subject fulfilling the diagnostic criteria of SS with SSA/SSB seropositivity and a low but positive focus score (i.e. 2 of 12), the subject displayed a normal unstimulated salivary flow rate and was negative for ocular problems as determined by the Schirmer’s test. Although two different serum samples taken 1 year apart from this SS subject were similarly positive for interferon-α autoantibodies, the exact onset of the anti-cytokine autoantibodies in this subject and other cases is not known. Potentially confounding this analysis is the recognition that SSA autoantibodies and other autoantibodies are often present over 18 years before clinical diagnosis of SS [48], and it is unclear when the anticytokine autoantibodies might arise. Nevertheless, it is tempting to speculate that after the initial onset of SS in this patient that induction of anti-interferon-α autoantibodies may have later dampened interferon signaling resulting in improved clinical features. As previously proposed, the production of certain cytokine autoantibodies may be caused by by-stander autoimmunization and/or be part of a natural feedback loop to decrease cytokine signaling in chronic inflammation [49, 50]. Recently, anti-interferon-α therapy has shown promising clinical results by reducing SLE disease activity [51, 52]. By extension, our unique findings that the one SS patient harboring naturally, partially neutralizing anti-interferon-α activity exhibited milder sicca symptoms is potentially consistent with the idea that blocking the interferon-α pathway might show efficacy for the treatment of SS. Future studies exploring whether anticytokine autoantibodies exist in saliva derived from the salivary gland and the cell/tissue origin of the interferons involved in autoimmunization may provide additional insights into the functional significance of these autoantibodies in SS.

## Abbreviations

ANA: antinuclear antibodies
DNTM: disseminated non-tuberculous mycobacterial
ENA: extractable nuclear antigen
HV: healthy volunteer
LIPS: Luciferase immunoprecipitation systems
LU: light units
PAP: pulmonary alveolar proteinosis
RF: rheumatoid factor
SD: standard deviation
SLE: systemic lupus erythematosus
STAT-1: Signal Transducer and Activator of Transcription-1
SS: Sjögren’s syndrome
